# The Ghana essential health interventions program: a plausibility trial of the impact of health systems strengthening on maternal & child survival

**DOI:** 10.1186/1472-6963-13-S2-S3

**Published:** 2013-05-31

**Authors:** John Koku Awoonor-Williams, Ayaga A Bawah, Frank K Nyonator, Rofina Asuru, Abraham Oduro, Anthony Ofosu, James F Phillips

**Affiliations:** 1 Regional Health Administration, Ghana Health Service, Upper East Region, Bolgatanga, Ghana; 2 Heilbrunn Department of Population and Family Health, Columbia University, New York, NY 10032, USA; 3 Ministry of Health, Ministries, Accra, Ghana; 4 Ghana Essential Health Interventions Program Regional Health Administration, Ghana Health Service, Upper East Region, Bolgatanga, Ghana; 5 Navrongo Health Research Centre, Ghana Health Service, Upper East Region, Navrongo, Ghana; 6 Policy Planning Monitoring and Evaluation Division, Ghana Health Service, Accra, Ghana; 7 Heilbrunn Department of Population and Family Health, Columbia University, New York, NY 10032, USA

## Abstract

**Background:**

During the 1990s, researchers at the Navrongo Health Research Centre in northern Ghana developed a highly successful community health program. The keystone of the Navrongo approach was the deployment of nurses termed community health officers to village locations. A trial showed that, compared to areas relying on existing services alone, the approach reduced child mortality by half, maternal mortality by 40%, and fertility by nearly a birth — from a total fertility rate of 5.5 in only five years. In 2000, the government of Ghana launched a national program called Community-based Health Planning and Services (CHPS) to scale up the Navrongo model. However, CHPS scale-up has been slow in districts located outside of the Upper East Region, where the “Navrongo Experiment” was first carried out. This paper describes the Ghana Essential Health Intervention Project (GEHIP), a plausibility trial of strategies for strengthening CHPS, especially in the areas of maternal and newborn health, and generating the political will to scale up the program with strategies that are faithful to the original design.

**Description of the intervention:**

GEHIP improves the CHPS model by 1) extending the range and quality of services for newborns; 2) training community volunteers to conduct the World Health Organization service regimen known as integrated management of childhood illness (IMCI); 3) simplifying the collection of health management information and ensuring its use for decision making; 4) enabling community health nurses to manage emergencies, particularly obstetric complications and refer cases without delay; 5) adding $0.85 per capita annually to district budgets and marshalling grassroots political commitment to financing CHPS implementation; and 6) strengthening CHPS leadership at all levels of the system.

**Evaluation design:**

GEHIP impact is assessed by conducting baseline and endline survey research and computing the Heckman “difference in difference” test for under-5 mortality in three intervention districts relative to four comparison districts for core indicators of health status and survival rates. To elucidate results, hierarchical child survival hazard models will be estimated that incorporate measures of health system strength as survival determinants, adjusting for the potentially confounding effects of parental and household characteristics. Qualitative systems appraisal procedures will be used to monitor and explain GEHIP implementation innovations, constraints, and progress.

**Discussion:**

By demonstrating practical means of strengthening a real-world health system while monitoring costs and assessing maternal and child survival impact, GEHIP is expected to contribute to national health policy, planning, and resource allocation that will be needed to accelerate progress with the Millennium Development Goals.

## Background

Ghana has a long experience with evidence-based health systems development. During the early 1990s, debate about practical means of achieving the World Health Organization’s goal of “Health for All by the Year 2000,” led the Ministry of Health to implement an experimental maternal and child health program in Kassena-Nankana District of the Upper East Region (UER), Ghana’s poorest region. The program, known as the “Navrongo Experiment” was based at the Navrongo Health Research Centre, which due to its past involvement in international public health research, had a Demographic Surveillance System (DSS) that regularly conducted continuous monitoring of mortality, morbidity, and fertility dynamics in this largely rural Sahelian area. By 1998, preliminary results of the Navrongo experiment had begun to demonstrate that the project would have an impact. In the initial five years, fertility declined by about a birth from a total fertility rate of 5.5 and the maternal mortality ratio declined by 40% [1-5]. By the end of project monitoring in 2003, childhood mortality was reduced by 68% in communities where nurses were based while levels remained relatively unchanged in comparison areas.

The program’s success was based on at least two key features. First, it offered life-saving services delivered in a convenient, low-cost, and effective manner. Estimates derived during the Navrongo experiment suggested that the program, if faithfully scaled up, would add only $2.92 per year per capita to the revenue budget to launch, and an additional $1.92 per capita to current spending to sustain over time. Second, through community mobilization activities — especially with men — the program built a climate of trust between community health workers and extended families. Whereas clinical workers are required to extract fees from parents at the time of care, the community engagement system enabled community-based workers to trust clientele to eventually reimburse the system for pharmaceutical costs, even if families lacked cash at the time of care. This “trust as insurance” system ensured that extended families could be trusted to support emergency health care costs. Moreover, community engagement overcame “gatekeeping” of women’s health-seeking behavior. When women and children become ill in profoundly gender-stratified societies like those of northern Ghana, they are often denied the timely provision of simple, life-saving interventions because their elder women or male relatives are reluctant to allow them to seek care immediately [2]. This problem is particularly constraining for family planning services. Through community meetings, peer education, and other interventions, the Navrongo model helped relax these constraints on women’s behavior.

Despite the success of the Navrongo Experiment, the policy relevance of results was questioned by many district, regional, and national program managers. To address this skepticism, the district health management team (DHMT) from the Nkwanta District of the Volta Region launched a replication trial of the Navrongo experiment [3]. Implementation research showed that immunization coverage, service volume, and family planning acceptance replicated the Navrongo model [4,5]. In 2000, in response to this demonstration, scaling up of the Navrongo model, now called the Community-based Health Planning and Services (CHPS) Initiative, was adopted as national policy [6].

### The CHPS initiative

The primary staff resource for Community-based Health Planning and Services

(CHPS) are nurses, termed community health officers (CHOs), who spend 18 months in training schools and carry out an additional six-month internship for developing community liaison skills. CHOs are provided with essential equipment and assigned to health posts where they live and conduct doorstep services. This involves treatment of malaria, acute respiratory infections, and diarrheal diseases termed integrated management of childhood illness (IMCI). CHOs also provide comprehensive childhood immunization and family planning care for oral, injectable, and barrier contraception. CHOs live and work in health posts built with donated materials and the labor of community volunteers, and they are provided with a motorcycle. As resources become available, health posts are often upgraded or reconstructed as permanent structures that replace makeshift community-provided facilities. Volunteers care for diarrheal diseases, but they are mainly health promoters and referral agents who balance nurse outreach to women with a focus on the information needs of men and organizational activities. To support their work, these volunteers receive a bicycle.

CHPS occupies the “ground level” of the health system. Both CHOs and community volunteers provide services at the doorstep and at community health posts. As in the rest of the Ghana Health Service (GHS), trained paramedics provide care at sub-district health centers, serving roughly six to 10 villages or 20,000-30,000 people, and clinicians provide surgical and other specialty care at district hospitals. Of the three districts– Garu-Tempane, Bongo, and Builsa – in the Ghana Essential Health Interventions Project (GEHIP) , Garu-Tempane lacks a hospital and medical coverage. Financial management and policy guidance is provided by a district health management team comprised of a District Director of Health Services and officers responsible for disease control, nursing, clinical operations, and nutrition. Supplemental funding for CHPS is sometimes provided by Regional Health Administration (RHA) resources, but uniform standards for such support is lacking.

The Navrongo experiment demonstrated the limitations of basing child survival programs on access to commodities and/or clinical care alone. In one of the three study areas, briefly trained, unpaid volunteers were deployed to refer cases and provide antipyretics, vitamins, and other non-prescription drugs. Over the short-term, child mortality actually rose in this area compared to a control area where no interventions were offered, other than those routinely offered by the GHS. Research subsequently showed that syndromic intervention by credible but poorly trained volunteer workers delayed parental health seeking for effective curative care [2]. Only when comprehensively trained and fully paid nurses were posted to these areas did child mortality begin to fall substantially [5]. This crucial lesson still has yet to be internalized by many international donors, many of whom continue to favor interventions based on the distribution of simple commodities or health promotion by untrained volunteers alone, eschewing more substantial health system interventions because they seem complicated and expensive [7].

Properly trained and equipped community health workers can have health equity effects. In the Navrongo experiment, nurse care offset the detrimental effects that low parental-educational attainment and relative household poverty had on immunization, health-seeking behavior, and child survival. Volunteer services had no comparable equity effects [8]. However, if nurse-provided community-based care was combined with health promotion activities of volunteers, family planning gained credibility and both fertility and maternal and child mortality declined. Thus, the combined approach was adopted as the organizational model for CHPS.

Ghana aims to expand CHPS to all communities by 2015 with finances provided largely through government resources, although there is no health-sector budget provision for the cost of launching CHPS. Additional support is provided by NGOs, district assemblies, and the global community. Facility costs, equipment costs, and special start-up investments are not routinely available. But, flexibility for financing these costs exists in the development sector. In particular, development revenues of the World Bank, the European Union, and some bilateral donors are committed to flexible revenue accounts managed by decision makers with the District Chief Executive and District Assembly development. Whereas policies of the “Sector Wide Approach” once provided flexible revenue to district health managers, all fiscal flexibility is now managed by district political authorities. This pool of resources is combined with the government of Ghana’s flexible financing as well as by communities in the form of material and volunteer labor. Taken as a common fund, this source of revenue provides crucial district development resources that are external to the health sector but could be used to finance the essential $2.92 per capita in CHPS start-up costs. Since only about $14 per capita is available for all health expenditures combined, any meaningful contribution to the $2.92 per capita represents a major catalytic investment in CHPS expansion [9]. However, district officials must decide to make and sustain this investment, despite competing demands on the development budget from other sectors.

Where CHPS leadership is well-articulated, district political commitment has directed resources to the $2.92 per capita incremental start-up costs. Exchanges between districts have been critical to demonstrating effective means of developing this commitment. By 2008, CHPS implementation had commenced in all of Ghana’s districts, but scale-up within districts had stalled or was incomplete nearly everywhere. CHPS, as it was originally envisioned, was reaching only 12% of Ghana’s households [4]. Where Regional Health Administration (RHA) support involved the financing of exchanges between districts, there was active engagement with political and development authorities. Routine discussion of CHPS at staff meetings led to a small investment in CHPS and generated pilot implementation zones within districts. These demonstration communities, in turn, were instrumental in establishing a process of CHPS implementation within a given district that was rapid and straightforward. The Nkwanta experience showed that proper introduction within a given district, with strategies for community engagement, could catalyze political and NGO investment in scale-up. Through peer-to-peer exchanges, district leaders who had implemented CHPS successfully were able to persuade those in other districts to do the same, but this “catalytic leadership” was hard to define programmatically and has not been instituted on a national scale [10]. Donor support for some aspects of CHPS expansion has been generous but has tended to support technical assistance and workshops rather than the political mobilization that seems necessary to transfer implementation capacity from one district to another.

The fundamental problem was that CHPS was originally conceived as a community-based trial focused on identifying the best way of delivering services and sustaining community engagement for primary health care, rather than a systems initiative that involved interventions for developing district and regional leadership. Research on CHPS was focused on identifying the best way of delivering services and sustaining community engagement for primary health care. However, scaling up CHPS is a district systems issue and requires improved capabilities in regional and district management, planning, budgeting, and resource development. This, in turn, requires political mobilization beyond the community level.

In addition, fidelity to the original CHPS model developed at Navrongo has dissipated with passing time — a scaling-up phenomenon noted elsewhere [11-13]. For example, the Navrongo model encouraged communities to construct health posts for CHOs from donated materials with volunteer labor. Construction of permanent facilities was meant to be a reward for this community activity. However, some district managers delayed nurse deployment until revenue became available for financing outside contractors to construct health posts. Consequently, construction has become a constraint to implementation rather than an incentive for community action. Using funds to hire outside contractors also substantially raised the potential cost of scaling up, creating a further disincentive for donors and others to support the project.

The package of services was also often incomplete and proven life-saving components were needlessly excluded from the regimen. For example, supervision of nurses and volunteers was inadequate in many districts and information systems were so cumbersome that they were useless to CHOs. Another problem was that district leadership often prioritized ambulatory clinical care of adults rather than building community and political engagement to encourage community-based preventive health services and early treatment of the leading causes of childhood morbidity. In addition, owing to official National Nurse Midwife Council objections, CHO training excluded emergency obstetric care — life-saving skills, such as the management of asphyxiation and haemorrhaging, and proven approaches to saving newborn lives. In CHPS zones that were as yet incomplete, IMCI services were often inaccessible because there was no CHO. Volunteers might have been able to provide some of these services, but since they were often poorly trained and supervised, the GHS did not allow them to provide antibiotic therapy.

Thus, despite evidence that community-based primary health care was scalable and affordable, health conditions remained needlessly poor. According to national statistics at GEHIP baseline, infant mortality was 50 per 1000 live births and under-5 mortality was 80 per 1000 person-years [12]. However, roughly comparable rates applied in the Upper East Region (UER), (46 per 1000 live births and 78 per 1000 person-years, respectively) even though this is the poorest part of the country. Research in progress suggests that the wider implementation of CHPS in a way that was faithful to the original Navrongo experiment largely explains this apparent paradox.

## Description of the intervention

### GEHIP – a solution to the challenges of CHPS

GEHIP is a quasi-experiment designed to test the proposition that a novel set of interventions could improve the impact of CHPS, accelerate its adoption by districts, and, thereby, improve the health and survival of children under 5. Its interventions are informed by a prior initiative in Tanzania, known as the Tanzania Essential Health Interventions Project (TEHIP), which developed and tested tools for evidence-based planning, resource mobilization, and district health system leadership. GEHIP is posited on the assumption that improved planning, resource allocation, and leadership will accelerate CHPS, improve CHPS functioning, and reduce mortality as a result. During the 1990s, TEHIP was shown to have significant effects on child health and survival in that country, but its main potential for contributing to Ghana was its success in scaling up. Within a brief period, TEHIP transformed national management training, planning, and resource mobilization in all 120 districts of the country [13]. In the case of GEHIP, the Tanzania district systems strengthening approach is augmented with frontline worker training, emergency referral systems development, and other health systems strengthening initiatives that, when implemented together, are posited to have synergistic effects on CHPS implementation. But, mainly, GEHIP has borrowed the TEHIP focus on district planning capacity, resources, and leadership development. By doing so, GEHIP aims to set the stage for Ghana to scale-up CHPS and replicate the success of Navrongo in every community of the country.

### Collaborating partners

In the UER, GEHIP interventions are led by Dr. John Koku Awoonor-Williams, the Regional Director of Health Services, and managed by a secretariat based at the UER/RHA. Nationally, GEHIP is supported by the Director General of the GHS and conducted in collaboration with the GHS Policy, Planning, Monitoring and Evaluation (PPME) Division, which develops national health policy. PPME also led the engineering of a new budgeting and planning technology known as the District Health Planning and Reporting Toolkit (DiHPART), based on a system originally developed in Tanzania by the Ifakara Health Institute (IHI) and the University of Dar es Salaam Computing Centre. The University of Ghana School of Public Health (UGSPH) and Columbia University’s Mailman School of Public Health (MSPH) contribute research and scientific technical expertise to the project. Scientists at the Navrongo Health Research Centre (NHRC), a field station of the GHS, conduct monitoring and evaluation research.

### The geographic scope of GEHIP

**Figure 1:**
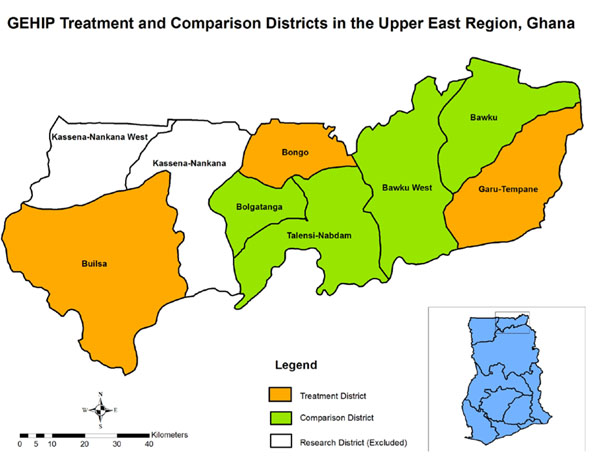
GEHIP intervention and comparison districts of the Upper East Region

Since so few districts are available for the selection process, GEHIP is a plausibility trial rather than a true experiment. Contrasting baseline conditions will require statistical adjustment at the close of the project[14]. GEHIP is being implemented in Builsa, Bongo, and Garu-Tempane districts of the UER in rural northern Ghana (Figure 1). Four contiguous UER districts have been purposefully selected as comparison districts. Two districts — Kassena-Nankana East and West — are the location of research projects of the Navrongo Health Research Centre (NHRC). Owing to the potentially confounding effects of successful NHRC trials of health service interventions, these special research districts are excluded from GEHIP [15]. The project was positioned in this challenging environment because policy deliberations about the relevance of success could be compromised if the context for solving problems was favorable to achieving positive results. Indeed, the dire circumstances and geographic isolation render the study areas to be challenging contexts for success of any kind. The treatment and comparison districts are ranked among the poorest 5% of Ghana’s 172 districts, with economies dominated by subsistence agriculture, low literacy, pervasive poverty, and per-capita income levels that are about a quarter of the level estimated for Ghana as a whole [16]. To ensure that GEHIP is focused on a challenging environment, the two research districts where the NHRC operates are excluded from GEHIP owing to unusually favorable health and survival conditions that have been induced by research initiatives in that locality.

### GEHIP interventions

#### Improving IMCI and related community-based services

National survey research and review of Navrongo long-term trends showed that neonatal mortality has declined more gradually than post-neonatal mortality. GEHIP has, therefore introduced the Save the Children “Saving Newborn Lives” intervention package for both nurses and volunteers [17]. This involves instituting procedures for promoting facility-based delivery, knowing the timing of delivery, providing immediate post-delivery follow-up for neonates that are born at home, providing “kangaroo mother care” training for mothers of premature neonates, and developing an emergency referral system that prevents delay in care when emergencies arise. Because IMCI services remained inaccessible in communities where CHPS has yet to be implemented, a program of training volunteers in antibiotic therapy and malaria treatment has been introduced that involves intensive supervision, referral services, and follow-up care. These interventions, together with in-service refresher training for all frontline workers, are aimed at strengthening the community-based service system.

#### The simplification of information systems

Procedures for data collection have been simplified with the elimination of gratuitous registers and forms in conjunction with the development and testing of a new health management information system (HMIS) known as the District Health Information Management System (DHIMS-2). Designed to support the decentralization of the health care system, DHIMS-2 improves the flow of information and supports the integration of health service operations. With the completion of GEHIP register simplification, DHIMS-2 addresses the need of community-based health care workers for simple and accessible information for supporting routine service delivery operations. Previously, cumbersome paper registers were required, along with tedious manual data aggregation procedures, requiring CHOs to spend copious amounts of time recording patient visits and registering insurance forms. Ghana’s efforts to expand access to its health insurance program only added to the information burden. A GEHIP baseline time-use study found that workers spent more time on paperwork than client care [18] and received no useful feedback or guidance from these efforts. GEHIP introduced a “simplified register” to condense the volume of registers from 27 to five. Taken as a set of interventions, these GEHIP activities aim to improve the quality, intensity, and access of primary health care.

#### Improving district leadership, management, planning, and political engagement

District health management teams require strengthened capabilities to make community-based care happen. These capabilities include management functions that foster community liaison and social mobilization, grassroots political engagement, volunteerism, gender-based communication, male outreach, etc. The GEHIP initiative aims to develop, test, and disseminate this package.

Budgeting and resource mobilization has also been missing in the CHPS implementation experience. Indeed, when managers are interviewed about the reasons for the failure of CHPS to scale- up, the most widely cited problem concerns resource constraints and lack of feasible strategies for solving the resource mobilization challenge. While budget lines exist for activities that frontline workers can implement, the cost of launching CHPS services, particularly developing practical means for DHMT to raise support for the construction of health posts where workers can live and work, has no GHS budget line. Therefore, this has been a key focus of the GEHIP program.

The GEHIP team entered into a partnership with counterparts in Tanzania who developed a project that combined a budgeting tool and an additional dollar per capita per year for five years. District managers were then able to use the tool to estimate the burden of disease (BoD) implications of investing that dollar in different programs. Research showed that this strategy enhanced the effective allocation of resources. With technical support from the Tanzanian PHIT Partnership team, their “PlanRep Toolkit” was re-engineered for trial by GEHIP as the District Health Planning and Reporting Toolkit (DiHPART). The implementation of DiHPART was designed to address the absence of a budget line for CHPS and the rational spending of health resources by districts. DiHPART enables district managers to allocate budget priorities according to their relative impact on the burden of disease. GEHIP also adds $0.85 per capita to district budgets per project year for DiHPART-guided programming.

**Figure 2:**
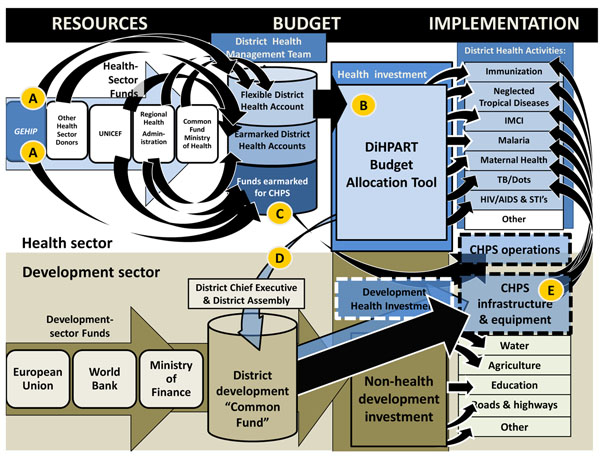
The flow of health and development resources to the health system.

GEHIP has also used DiHPART to conduct broader training sessions with district and sub-district officials. Shortages in trained leadership for district operations, especially in the areas of planning, implementation, and community engagement are undermining Ghana’s efforts to strengthen its health system and foster CHPS expansion. Figure 2 illustrates the budgeting and finance system that governs the flow of resources to primary health care. Two general sectors of funding are available — the health common fund, portrayed at the top of the diagram, and components of the development budget that can be allocated to health, shown at the bottom. As the figure shows, foreign assistance revenue is allocated by the development and political system rather than the health sector team. As a result, prior to GEHIP, most development revenue was earmarked for agriculture, education, roads, and other investments. While there is revenue earmarked for health, it explicitly omits CHPS start-up costs, in particular, health post construction. GEHIP addresses this resource gap by investing part of the $0.85 in flexible financing in the cost of interim facilities, community gatherings for celebrating the completion of construction milestones, and exchanges between health workers and grassroots politicians. By facilitating participation of chiefs, elders, opinion leaders, and politicians, GEHIP sets the stage for the allocation of development resources for CHPS facility costs and builds political will for resources to flow to the health sector.

As Figure 2 shows, financial support for the health sector is rather complex. District health management teams direct the utilization of flexible health accounts, earmarked funds for specific health service activities and earmarked funds for CHPS from external resources. Of the CHPS funding, UNICEF provides support for primary health care equipment and pharmaceutical supplies. This support enables UER districts to equip nurses for community-based health care operations. Of the $0.85 GEHIP provides per capita per year to DHMT. About half of this is earmarked for CHPS start-up activity while half is used to supplement the common fund (“A” in Figure 2). DiHPART facilitates the allocation of flexible funds that are received from the UER-RHA, the Ministry of Health, and GEHIP (“B” in Figure 2). Most funds that are available for CHPS are earmarked for equipment or for supporting ongoing community-based service activities, with no funding available from the health sector for start-up activities such as CHPS facility construction (“C” in Figure 2). However, DiHPART has mobilized the financial commitment of the district development sector to CHPS. This, in turn, has fostered district development investment in CHPS construction and procurement (“D” in Figure 2). This expanded resource for CHPS start-up costs has addressed the critical gap in health financing in GEHIP treatment districts and it explains their rapid implementation of CHPS and the outcomes associated with community-based primary health care (“E” in Figure 2).

**Figure 3:**
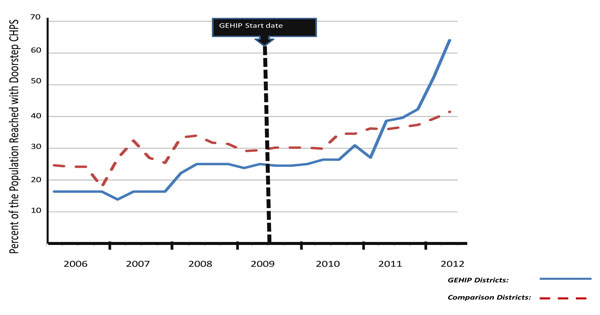
Time trend in the percent of the population reached by CHPS services in GEHIP treatment and comparison districts, January 2006-June 2012.

DiHPART has also become a tool for demonstrating the value of district development investment in CHPS. Prior to DiHPART, district managers lacked revenue for starting CHPS operations and practical tools for interacting with local politicians and development officers who had control of development resources for bridging funding gaps. The toolkit helped GEHIP district teams interact with counterparts with simple to understand visual diagrams of the value of health investment. The combination of leadership demonstration activities, community interaction, and DiHPART demonstrate the value of CHPS on scale-up in the three project districts, as Figure 3 shows. In 2009, coverage of CHPS in treatment districts was lower than in comparison districts. Today, after two years of GEHIP, coverage in treatment districts is now reaching 68% of the population, or roughly double the level of coverage in comparison districts. Indeed, projecting the rate of change in coverage suggests that GEHIP will have completed the scale-up process within another year in treatment areas; total coverage, however will require over six years of effort in comparison areas at the present rate of progress. Clearly, the combined effects of leadership training, catalytic investment, political engagement, and evidence-based budgeting are solving the CHPS start-up problem.

## Evaluation design

Both quantitative and qualitative research methodologies will be used to evaluate the impact of GEHIP.

### 

#### Assessing impact

To adequately power the sample for unbiased estimates of posited changes in child survival, it is estimated that a total sample size of 5,741 women is required. However, assuming response/attrition of 10%, GEHIP increased the planned sample size to 6,300 respondents to ensure an adequate analysis base despite possible sample loss. GEHIP controls for baseline differences in background characteristics with “difference-in-differences” estimates of program impact [19] as the average treatment effect (ATE) given by:

Y describes a health outcome such as the under-5 mortality rate or another of the indicators appearing in Table 1, the subscript *t* refers to measurements of health outcomes at baseline, t′ refers to measurements of health outcomes at the end of the project, G indexes GEHIP localities, and C indexes comparison localities.

**Table 1:** Core Maternal, Newborn and Child Health Impact Indicators

Category	Indicator
**Mortality rates (by gender of child)**	• Neonatal mortality rate • Infant mortality rate • Under-5 cumulative mortality rate (q5 ) • Estimated under-5cause specific mortality rate for malaria, acute respiratory infections

**Maternal Health Behavior (last live birth)**	• Percent of women who made their first ANC visit before the fourth month • Percent of pregnant women who attended four or more ANC visits • Percent of pregnant women who attended postnatal care visit • Percent of women who deliver in a health facility • Proportion of C-sections among last born.

**Newborn Health**	• Percent of newborns attending postnatal care visits • Percent of newborns breastfed within 1 hour of birth • Percent of women that report having practiced at least three ENC behaviors

**Under-5 child health**	• Percent episodes of diarrhea in children under 5 treated with ORS + zinc • Percent episodes of cough/pneumonia in children under 5treated with antibiotics • Percent of febrile malaria episodes among children under five treated within 24 hours of onset

**Family planning and fertility**	• Contraceptive prevalence rate • Unmet need for family planning • Age specific and total fertility rates

Data from the household surveys on health status can be linked to facility data and health system readiness checklists to allow the project to determine whether changes in health outcomes are attributable to health systems processes. Multilevel hazard models of under-5 survival in the 60 sample GEHIP clusters will permit estimation of the effect of health system strengthing on child survival, adjusting for the potentially confounding effects of household characteristics.

If GEHIP replicates the full effect of the Navrongo model, then its success in scaling up CHPS will reduce childhood mortality by half in three years.

A limitation of the impact evaluation strategy is the timing of the endline survey, scheduled for mid-2014. To gauge the full impact of the Figure 3 CHPS coverage success, the survey should be conducted approximately three years after July 2013, when coverage will be complete. While results of a survey in 2014 may establish statistical significance of results, the more important question concerns the potential impact of achieving a new global goal for impoverished rural settings: “University Health Coverage” comprised of doorstep primary health care for all, comprehensive preventive health services, cost-free emergency referral, and care for mothers and children.

#### Assessing organizational context, inputs, processes, and outcomes

The quality of administrative data and HMIS may be improved by GEHIP activities, resulting in spurious changes in health systems indicators in intervention districts relative to comparison districts. For example, drug shortages may appear to be more common in intervention areas not because they are actually increasing but because facilities in intervention areas are better equipped to report such shortages. To account for this possibility, the project conducts recurrent facility surveys in intervention and comparison localities at all levels of care, including CHPS compounds.

GEHIP monitors systems inputs and outputs and processes in intervention and non-intervention areas through administrative data collection, HMIS, qualitative appraisal, and facility-based surveys. The project will also rely on the CHPS monitoring database, a tool for documenting the national state of CHPS implementation.

GEHIP has adapted procedures that were developed for the qualitative appraisal of CHPS implementation to the study of GEHIP implementation [20]. Focus groups and in-depth interviews are conducted among community stakeholders, frontline workers, supervisors, and district managers. To facilitate process documentation, a knowledge management scheme compiles qualitative data and narrative summaries of project implementation experience, problems that arise, and implementation lessons that are learned. A series of journalistic notes has been launched to capture salient episodes from this experience [21].

#### Other contextual factors

Activities in both the intervention and non-intervention districts are occurring which the project lacks control. For instance, all three northern regions of Ghana are target districts for interventions by UNICEF and other donors. Since these priority interventions will take place in both treatment and comparison districts, donor priorities may contaminate the GEHIP intervention. GEHIP collects contextual information on external activities that may impact the primary outcomes of interest. Of particular significance is the recent parliamentary decision to split districts into smaller political units. At the onset of the project, there were nine functioning districts; by mid-2013 there will be 13. Data compiled from each sample cluster will include indicators of exposure to investments of external agencies and indicators of changes in political boundaries so that multilevel analyses can adjust for their potentially confounding effects and exposure to project interventions is appropriately attributed to sample survey clusters.

#### Economic evaluation

Economic evaluation of the project will assess the unit cost associated with net health benefits that accrue from GEHIP expenditures. Cost and expenditure data are being gathered at each level of the system to allow for estimation of benefits resulting from supplementary expenditures in intervention districts. Efforts are directed to assessing external investment in regional programs, with particular attention to expenditures of UNICEF and other international agencies.

## Implementation challenges and lessons learned

Table 2 summarizes challenges to the effective provision of primary health care in rural areas of Ghana and GEHIP implementation lessons about feasible strategies for addressing them. At baseline, neonatal and maternal mortality rates were unacceptably high, but the rapid training of frontline workers proved to be inexpensive, operationally feasible, and potentially effective in reducing maternal and neonatal mortality. Moreover, a pilot referral system utilizing tri-car ambulances has been implemented in one GEHIP district and information systems have been reformed through the adoption of the simplified register system.

**Table 2:** GEHIP PHIT implementation progress: success, challenges, adaptations.

***Successes***
***Development of a toolkit for budgeting and finances.*** With technical assistance from the University of Dar es Salaam Computing Centre and the Ifakara Health Institute, GEHIP re-engineered Tanzania’s PLANREP budgeting toolkit for Ghana. This involved utilizing Navrongo Health Research System generated burden of disease data and Government of Ghana accounting procedures to develop a model for translating budget plans into visualized data on their burden of disease implications: The District Health Planning and Reporting Toolkit (DiHPART). ***Development of simplified HMIS data capture and data utilization procedures*** GEHIP provided direct support to a reform of the national “District Health Information Management System Version-2” by providing community-level components of the reformed system. This involved streamlining registers, testing their application, revising content, and developing feedback tools, data visualization, and supervisory leadership mechanisms for improving the use of HMIS for decision-making. ***Mortality auditing.*** All frontline workers have been trained to produce simple-to-interpret narrative reports on all known maternal and neonatal deaths. A medical review panel has been constituted to conduct weekly reviews of these audits. ***Training of frontline workers in emergency management*** All frontline workers were trained to refer deliveries and care for newborn needs. Missing elements of emergency management were identified by GEHIP mortality audit scheme with training instituted in response to problems. ***Improved collaboration and co-financing of community based primary health care by district and local government.*** All district managers were trained in CHPS implementation and equipped to use DiHPART for orienting district political leaders to the benefits of CHPS investment. Improved understanding of the health benefits of CHPS has catalysed incremental funding for implementing community-based care. CHPS coverage accelerated as a consequence.

**Challenges**

***DiHPART.*** Trial of the system demonstrated that categories used for data visualization are inconsistent with decision-making options. Also, changes in the national accounting system are not yet reflected in the DiHPART tool. DiHPART requires re-engineering based on lessons learned. ***Cash flow and planning***. DiHPART assumes that the district level common fund is available for managers to allocate according to plans. Long delays in the allocation of Common Fund revenue challenge that assumption. Actual expenditure patterns differ from budget parameters because of unpredictable flow of essential revenue. ***Fidelity to proven operational models.*** Implementation of the National Health Insurance System (NHIS) has dysfunctionally shifted the focus of care to clinic-based services, detracting from outreach and operational strategies that have been proven to work. ***Excess mortality*** Audits have revealed excess neonatal mortality from asphyxia, and maternal mortality from convulsions, and haemorrhaging. ***Effective supervision at the community level.*** Research shows that supervisory field encounters are the main factor affecting community health worker performance. However, NHIS reimbursement policies reimburse supervisory staff for clinical services rendered. Supervisory field work has diminished. ***Timing of Systems Changes.*** As Figure 3 shows, GEHIP has taken time to implement: changing the leadership system, accelerating the flow of resources from the development sector, and the implementing community health services has taken 18 months of project time that impacted on CHPS coverage in project Years 2 and 3. The full child survival impact of GEHIP will be realized well after the project is completed.

**Adaptations**

***DiHPART.*** While DiHPART was conceived as a resource allocation tool, its value to GEHIP has shifted somewhat to resource development. Visualizing the health benefits of investment in CHPS has facilitated district dialogue about ways for development revenue to bridge critical resource gaps. The absence of budget lines for CHPS start-up costs prevents the expansion of community-based primary health care, leadership training, field demonstration, and DiHPART have been combined into a paradigm for multi-sectoral investment in CHPS expansion. ***Excess mortality.*** High neonatal and maternal mortality from preventable causes has fostered strategic planning about training needs, referral systems development, and information systems reform. Research findings and plans have been translated into fund-raising initiatives that are successfully augmenting GEHIP with resources for addressing excess mortality. ***Fidelity to proven CHPS strategies.*** GEHIP has been a mechanism for systems diagnosis, documentation, and national dialogue about implementation lapses in the national CHPS program. Simple to implement corrective measures that are instituted by GEHIP are developing treatment districts into a national learning platform for health systems development. The UER, in turn, is becoming a learning region for guiding national strategies for achieving universal health coverage, accessible care, and comprehensive community health services. ***Timing of Systems Changes.*** The national CHPS monitoring system lapsed in 2008 with the conclusion of external funding arrangements. In response, GEHIP has developed reformed CHPS coverage monitoring tools that enable the project to conduct longitudinal observation of the scale-up of CHPS implementation (as illustrated by Figure 3). New monitoring tools, integrated into DHIMS-2, will enable the GHS to have real-time access to information about systems development progress and lapses.

The GHS PPME Division has committed to merge the simplified GEHIP registers with its new DHIMS-2 system and then scale the combined system up nationally, once technical problems with the existing tool are resolved.

Implementation research has also been crucial to the process of scaling up. For example, problems arose during the piloting of the ambulance based emergency referral program described above. A bilateral donor provided Ghana with several hundred fuel efficient three-wheel “tri-cars,” several of which the GHS consigned to GEHIP for the pilot trial. However, tri-car ambulances designed for Asian urban conditions were highly unsuitable to the terrain of northern Ghana. Problems also arose during a trial of procedures for organizing and streamlining communication between remote health posts, sub-district health centers, and district hospitals using mobile phones. The study itself was successful but [22] advanced engineering “m-health” concepts, involving the extension of automated voice communication to mothers, was found to be less effective than simple-to-implement face-to-face voice communication between mothers and health workers [23,24].

GEHIP has encountered other problems with DiHPART implementation. The tool assumes that “interventions” are grouped by disease syndromes or broad categories of activities that are complex, involving a wide range of investment options of varying degrees of efficacy or efficiency. IMCI, for example, is a complex array of community, clinical, and hospital-based activities. Disaggregating IMCI as a DiHPART parameter would assist DHMT in determining which IMCI investments make the most sense, given the resource environment. In revised form, DiHPART would visualize the burden of disease implications of more detailed health expenditure options.

DiHPART implementation within districts has also proven to be challenging, owing to persistent staff turnover and hardware issues. Analysis of output from DiHPART has been compromised by software limitations, including its inability to provide for the management of actual cash flow. The lack of expenditure tracking capabilities impedes the administrative requirement of comparing actual spending to the planned budget. While district managers utilize DiHPART to plan activities, the system is not yet an accounting tool. As these issues are resolved, the tool is likely to be used at national and regional levels, enabling health plans to be based on scientific evidence and, therefore, more effective.

## Conclusion

In summary, GEHIP is a quasi-experimental study of a program designed to accelerate the scale- up of the Navrongo experiment — one of the most effective health development experiments conducted in Africa [25]. GEHIP supplements the provision of effective primary health care strategies with leadership training, field demonstration, improved budgeting, and resource mobilization. By means of these interventions, GEHIP aims to enhance health equity, mitigate social and monetary health care costs, foster parental health-seeking behavior, and improve maternal and child survival. Training has been designed to expand access to life saving technology that can reduce neonatal, infant, and childhood mortality.

By focusing on community health activities alone and neglecting the larger political and development context, CHPS was unable to mobilize needed district leadership, budgeting, finance, and planning components. And, even where it went to scale, its impact was often impaired by the absence of capabilities to manage emergencies and save newborn lives. GEHIP aims to correct these problems with district systems strengthening activities and improving primary health care. Preliminary research results, illustrated in Figure 3, show that interventions are having their intended impact on the pace of CHPS scale-up. If, as expected, this success translates into an impact on child mortality, GEHIP will provide a critically needed focus for national efforts to develop primary health care and lessons for international health experts as well.

## List of abbreviations used

BoD: Burden of Disease; CHO: Community health officer;CHPS: Community-based Health Planning and Services; DHIMS-2: District Health Information Management System; DHMT: District health management team; DiHPART: District Health Planning and Reporting Toolkit; DSS: Demographic Surveillance System; GEHIP: Ghana Essential Health Intervention Project; GHS: Ghana Health Service; HMIS: Health Management Information System; IHI: Ifakara Health Institute; IMCI: Integrated management of childhood illness; MSPH: Mailman School of Public Health; NGO: Non-governmental organization; NHRC: Navrongo Health Research Centre; PPME: Policy, Planning, Monitoring and Evaluation; RHA: Regional Health Administration; TEHIP: Tanzania Essential Health Interventions Project; UER: Upper East Region; UGSPH: University of Ghana School of Public Health.

## Competing interests

The authors declare that they have no competing interests.

## Authors’ contributions

JA, AB, and JP participated in the design and coordination of the study and in the drafting of the manuscript. AB is the GEHIP Director of Research. FN directed the design and development of the DiHPART initiative and collaborated with AN in the write-up of the section of the paper on DiHPART. RA directs implementation activities and contributed to the implementation section of the manuscript. AO collaborates with JP and AB on research design and implemetnation issues. JP conceived of the study and participated in its design and coordination. All authors read and approved the final manuscript.

## Declarations

This article has been published as part of *BMC Health Services Research* Volume 13 Supplement 2, 2013: Improving primary health care to achieve population impact: the African Health Initiative. The full contents of the supplement are available online at http://www.biomedcentral.com/bmchealthservres/supplements/13/S2. Publication of this supplement was supported by the African Health Initiative of the Doris Duke Charitable Foundation.
